# AI-Assisted Dynamic Port and Waveform Switching for Enhancing UL Coverage in 5G NR

**DOI:** 10.3390/s25185875

**Published:** 2025-09-19

**Authors:** Alejandro Villena-Rodríguez, Francisco J. Martín-Vega, Gerardo Gómez, Mari Carmen Aguayo-Torres, José Outes-Carnero, F. Yak Ng-Molina, Juan Ramiro-Moreno

**Affiliations:** 1 Communications and Signal Processing Lab, Telecommunication Research Institute (TELMA), E.T.S. Ingeniería de Telecomunicación, Universidad de Málaga, Bulevar Louis Pasteur 35, 29010 Málaga, Spain; 2 Cloud and Software Services at Ericsson, C. Severo Ochoa 55, 29590 Málaga, Spain

**Keywords:** deep reinforcement learning, waveform switching, 5G

## Abstract

The uplink of 5G networks allows selecting the transmit waveform between cyclic prefix orthogonal frequency division multiplexing (CP-OFDM) and discrete Fourier transform spread OFDM (DFT-S-OFDM) to cope with the diverse operational conditions of the power amplifiers (PAs) in different user equipment (UEs). CP-OFDM leads to higher throughput when the PAs are operating in their linear region, which is mostly the case for cell-interior users, whereas DFT-S-OFDM is more appealing when PAs are exhibiting non-linear behavior, which is associated with cell-edge users. Therefore, existing waveform selection solutions rely on predefined signal-to-noise ratio (SNR) thresholds that are computed offline. However, the varying user and channel dynamics, as well as their interactions with power control, require an adaptable threshold selection mechanism. In this paper, we propose an intelligent waveform-switching mechanism based on deep reinforcement learning (DRL) that learns optimal switching thresholds for the current operational conditions. In this proposal, a learning agent aims at maximizing a function built using available throughput percentiles in real networks. Said percentiles are weighted so as to improve the cell-edge users’ service without dramatically reducing the cell average. Aggregated measurements of signal-to-noise ratio (SNR) and timing advance (TA), available in real networks, are used in the procedure. In addition, the solution accounts for the switching cost, which is related to the interruption of the communication after every switch due to implementation issues, which has not been considered in existing solutions. Results show that our proposed scheme achieves remarkable gains in terms of throughput for cell-edge users without degrading the average throughput.

## 1. Introduction

The 5G 3GPP specifications have brought a great degree of flexibility to cover a wide range of use cases, scenarios, and propagation conditions. One of the major enhancements was the addition of the millimeter-wave band (mmW), which allows increasing bandwidth and throughput, but it complicates uplink (UL) transmission due to the higher path loss. The UL power control mechanism aims at compensating for the path loss and shadowing, guaranteeing a target received power level at the base station (BS). However, the differences in the path losses of different users involve different power amplifier (PA) operating conditions, with some of them working at the linear region and others in the non-linear region. To handle the diverse PA operating and propagation conditions, 5G considered two UL waveforms: cyclic prefix orthogonal frequency division multiplexing (CP-OFDM) and discrete Fourier transform spread OFDM (DFT-S-OFDM), which is also known as single-carrier frequency division multiplexing (SC-FDM) and as transform precoding in the literature. This dual-waveform approach allows a dynamic selection of the most appropriate waveform for the user equipment (UE) working conditions [1]. The former waveform leads to higher throughput when the PAs operate in their linear region and better performance of multiple-input multiple-output (MIMO) techniques, whereas the latter leads to a smaller peak-to-average power ratio (PAPR). This reduction in PAPR is of paramount importance when non-linear PAs are considered, since it reduces the out-of-band emission (OOBE) and non-linear distortion. As shown in preliminary studies [2,3], in the presence of non-linear PAs and UL power control, DFT-S-OFDM leads to higher throughput compared to CP-OFDM for UEs placed at distant locations from its serving BS, whereas CP-OFDM offers higher throughput for the rest of UEs. The fact that the OOBE is smaller with DFT-S-OFDM implies that it is possible to transmit with a higher power than with CP-OFDM while fulfilling the same transmission spectral mask. This is especially relevant at mmW bands, where the high path loss significantly reduces the UL coverage. Hence, the 3GPP NR specifications define a maximum transmit power of DFT-S-OFDM, which is between 1.5 and 2.5 dB greater than that of CP-OFDM, depending on the bandwidth and constellation [1]. This higher transmit power extends the cell coverage when DFT-S-OFDM is used, which can potentially increase the lower percentiles of the throughput distribution in a cell. In addition, the introduction of reduced-capability (RedCap) devices for industrial networks exacerbates these aforementioned issues in the UL due to the related simplifications in their radio frequency and baseband capabilities [4]; therefore, motivating further the need for optimal waveform-switching solutions.

Despite the potential of waveform switching to improve UL performance, only a few works have investigated this issue. In [2], it was shown that there is a crossing point between CP-OFDM and DFT-S-OFDM in terms of throughput versus distance when a non-linear PA is considered. Then, a switching mechanism between both waveforms based on the distance was suggested to maximize the user throughput under adaptive modulation and coding (AMC). The performance of DFT-S-OFDM was compared in [5] with an improved version of CP-OFDM that uses clipping and filtering to reduce its PAPR. It can be concluded from this work that the optimum switching point depends, among other factors, on the number of allocated physical resource blocks (PRBs). Another method, which further reduced PAPR and led to reduced OBBE was presented in [3], which consists of a modification of the DFT-s-OFDM waveform, adding two blocks to perform frequency domain spectral shaping and extension. However, this requires changes in the standard, and does not remove the need to perform a waveform switching. The work presented in [6] also proposed a switching mechanism, but in this case, the switching is triggered by the difference between the measured packet error rate (PER) with a non-linear PA and the ideal PER that is predicted considering a theoretical model with a fully linear PA. If the difference in these PER measures is above a given threshold, DFT-S-OFDM is selected; otherwise, OFDM is used. A more feasible switching mechanism, which is based on the estimated SNR, was proposed in [7]. The received SNR is compared with some thresholds to select the waveform that maximizes the throughput with AMC under non-linear PA. Later, in [8], this approach is extended to consider MIMO. Here, depending on the SNR, it is selected CP-OFDM with either one or two layers, or DFT-S-FDM with a single layer.

All of these aforementioned works compare a given metric, i.e., distance towards the BS, PER difference between linear and non-linear case, or SNR, with some thresholds that are computed offline based on simulations for some specific setting. Therefore, these methods might fail to adapt to changes in the scenario. In addition, these works do not consider the penalty that exists in real 5G networks when a waveform switch is triggered. The 3GPP specifications impose a guard time where the UE cannot transmit after a waveform switch to allow the device to prepare for this change. Ignoring such guard time in the switching mechanism might lead to a ping-pong effect that severely degrades the achievable throughput.

To solve this important issue, a waveform-switching mechanism to optimize the power consumption of the UE was proposed in [9]. This invention, named dynamic port and waveform switching (DPWS), relies on the transmission of either radio resource control (RRC) configuration messages or a bandwidth part (BWP) switch to change the UL waveform. DPWS implements a mechanism based on thresholds and counters to avoid the aforementioned ping-pong effect that degrades performance. Nevertheless, the invention does not specify how to optimize such thresholds to maximize a given metric.

Traditional optimization methods often struggle with the dynamic and complex nature of 5G networks. These networks are characterized by highly variable user behavior, changing radio channel conditions, and a massive number of interconnected devices. This complexity creates a system where a single static set fails to maximize performance. Deep reinforcement learning (DRL) is uniquely suited for this environment because it is designed to solve problems where an agent must make a sequence of decisions in an uncertain environment to maximize a cumulative reward. The great potential of DRL for 5G arises from its ability overcome the complex interplay between network metrics, offering real-time inference capabilities [10]. Instead of relying on pre-programmed rules, a DRL agent learns a policy that adapts to a constantly changing network. This allows it to make decisions that are not only reactive to current conditions but also proactive, anticipating future changes based on its learned experience. Unlike traditional convex optimization-based approaches, DRL frameworks optimize a reward function by learning a fitting policy for a specific problem. Such a reward can be devised to optimize multiple, even competing, objectives simultaneously. The suitability of DRL for this problem comes from its ability to learn a policy by interacting with the system, applying changes, and observing the effects, rather than relying on ground-truth data. In addition, 5G networks generate vast amounts of data from various sources. DRL can effectively process this high-dimensional input to extract meaningful insights and make informed decisions, which would be challenging for simpler algorithms.

Thanks to these benefits, DRL has been recently applied to optimize a wide range of performance metrics and scenarios. In [11], DRL is applied in energy harvesting device-to-device communication scenarios to maximize the throughput subject to age of information (AoI) constraints. The task offloading and resource allocation problem in space-air-to-ground networks is addressed in [12], where a DRL framework built with graph neural networks for feature extraction is proposed. A resource allocation problem that considers the fairness of different users in terms of delay is considered in [13], where a DLR agent optimally selects the parameters of a modified largest weighted delay first scheduling algorithm. DRL is also applied to increase the fairness of 5G networks in [14], but in this case, it is considered the mobility management problem. Due to the aforementioned issues, the UL optimization of 5G networks has also been a major application scenario of DLR approaches. In [15] DRL is used to determine an optimal user clustering for non-orthogonal access that maximizes the throughput, whereas in [16,17], DRL is used to determine the optimal UL control commands to maximize the throughput and reduce delay, respectively. Nevertheless, as far as the author’s knowledge the problem of waveform switching as not been addressed with DRL yet.

In this paper, we present a novel solution, named artificial intelligence-assisted dynamic port and waveform switching (AI-DPWS), whose objective is to find the optimal values for the SNR threshold and the appropriate SNR hysteresis that maximize the cell’s performance in terms of UL throughput. Dynamic adjustment is made automatically based on real-time gNB measurements, thus adapting the optimal thresholds to varying conditions of the environment, which is in clear contrast to existing approaches for waveform switching in the literature, e.g., [2,6,7].

More specifically, the contributions of the present work can be summarized as follows:

- We propose a DRL-based mechanism named AI-DPWS that selects the optimal values for the SNR threshold and the appropriate SNR hysteresis that maximize the cell’s performance in terms of UL throughput. AI-DPWS aims to improve the throughput of the cell-edge UEs, i.e., those associated with a lower percentile of the cell´s throughput cumulative distribution function (CDF), without sacrificing the average throughput of the cell. This is possible thanks to the designed reward function, which encapsulates such a trade-off. Therefore, this approach increases the system fairness in terms of throughput, which is a paramount metric [13,14,18], with no penalty in terms of average throughput.
- The agent is trained using key performance indicators (KPIs) that are available in real-world networks, such as histograms of the throughput, UL SNR, and timing advance (TA), which are collected during real-life network operation. The aim of considering these realistic metrics is to offer a solution that can be deployed in real cells. To this end, a realistic 5G simulator has been developed. As detailed in Section 2, this simulator accounts for UL power control, propagation conditions, PA non-linearity, 5G compliant physical layer processing, and switching cost.
- The performance gains of the proposed method are shown with numerical results, confirming that the proposed method increases the performance of cell-edge users without sacrificing the average throughput.

## 2. System Model

Each UL transmission at the mmW band can use either a CP-OFDM or a DFT-s-OFDM waveform as indicated by the BS either by a BWP switch or a RRC reconfiguration message [19]. We investigate a link between a single cell and a single user in the UL direction, i.e., without interfering with UL transmission, considering the 3GPP specifications for the physical and medium access control (MAC) layers of 5G.

**Figure 1:**
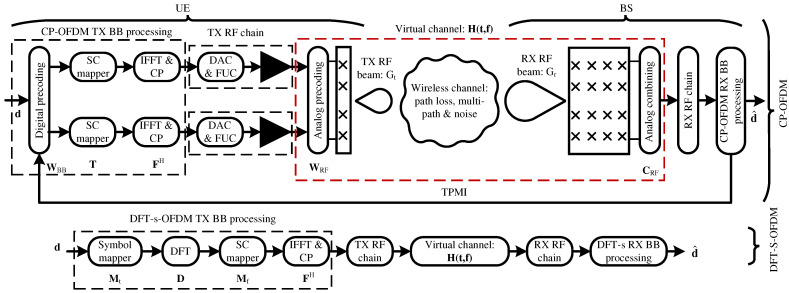
Block diagram of the communication system model for CP-OFDM and DFT-s-OFDM transmission. The diagram at the top side represents the transmitter (i.e., UE) and receiver (i.e., BS) for CP-OFDM with two ports. The bottom side diagram represents the case of the DFT-s-OFDM transmission. The transmit RF chain has the digital-to-analog converter (DAC), frequency up-converter (FUC), and PA. The virtual channel blocks include the effect of transmit and receive RF beams and multi-path propagation. The PAs are represented as a black triangle.

Figure 1 shows the end-to-end communication system model for both CP-OFDM and DFT-s-OFDM. The diagram at the top side represents the transmitter (UE) and receiver (BS) for CP-OFDM with two ports, while the bottom side corresponds to DFT-s-OFDM transmission. The transmitter chain starts with channel coding and symbol mapping, followed by waveform-specific processing. In CP-OFDM, symbols are precoded across ports, transformed to the time domain by an IFFT, and protected with a cyclic prefix. In DFT-s-OFDM, symbols first pass through a DFT precoder before subcarrier mapping and time-domain conversion. In both cases, the resulting samples are passed through the RF front-end, which includes the digital-to-analog converter (DAC), the frequency up-converter (FUC), and the power amplifier (PA), depicted as a black triangle. The virtual channel block models the effect of analog transmit and receive beams together with multi-path propagation. At the receiver side, the RF chain applies analog combining and down-conversion, after which the corresponding baseband demodulation (CP-OFDM or DFT-s-OFDM) is performed. This organization highlights that the two waveforms differ in their baseband processing stages, while they share the same RF and propagation stages. As it is illustrated in Figure 1, the UE and BS use a hybrid precoding architecture [20] where the precoding is divided into base-band (BB)—i.e., digital—and radio frequency (RF)—i.e., analog—domains. We consider passive phased antenna array panels at the UE and BS sides, each able to synthesize an aligned RF beam at the transmit and receive sides, whose gains are modeled as $G_{t}$ and $G_{r}$, respectively. The transmit and receive beams are synthesized with analog precoding, $W_{\mathrm{RF}}\in C^{N_{t}\times N_{p}}$, and combining, $C_{\mathrm{RF}}\in C^{N_{t}\times 1}$ matrices, being $N_{t}$ and $N_{r}$ the number of physical transmit and receive antennas. $N_{p}$ stands for the number of ports at the transmitter side, which represents the number of RF chains, which are also called logical antennas. This is due to the fact that the channel matrix that can be estimated at the receiver using sounding reference signals (SRS) is a virtual channel that includes the analog precoding and combining matrices, i.e., $H\left(t,f\right)\in C^{1\times N_{p}}$, since it is considered a single-port receiver for the BS in this work. Therefore, MIMO techniques in the digital domain consider the virtual matrix whose dimension is smaller than the channel matrix. Both DFT-S-OFDM and CP-OFDM schemes use beamformed demodulation reference signals (DMRS) to perform channel estimation for symbol detection (i.e., equalization). DMRS signals are digitally precoded if several ports are considered. Nevertheless, we also consider the periodic transmission of sounding reference signals (SRS), which are not digitally precoded, to estimate the SNR for waveform selection over the employed analog beam.

The BB transmission chain uses the low-density parity-check codes (LDPC) for the physical uplink shared channel (PUSCH). The proposed system allows selecting between two different UL waveforms: (i) DFT-S-OFDM waveform with 1 antenna port and 1 data layer, and (ii) CP-OFDM waveform with 2 antenna ports and 1 data layer. It is worth noting that the DFT-S-OFDM scheme only makes use of one antenna port. This design decision was taken due to the moderate to low performance of DFT-S-OFDM with MIMO [21,22,23,24,25] in the digital domain. Note that DFT-S-OFDM remains compatible with analog beamforming, benefiting from its directionality and increased link performance.

It is considered adaptive modulation and coding (AMC), therefore information bits are coded by the LDPC encoder and mapped into constellation symbols according to the MCS selected by the BS after SRS transmission. The vector of constellation symbols is represented as $d\in C^{1\times N_{d}}$ in Figure 1, where $N_{d}$ represents the number of allocated resource elements (RE) in a given slot.

In the case of CP-OFDM waveform, the constellation symbols are transformed via a precoding matrix $W_{\mathrm{BB}}\in C^{N_{p}\times 1}$ where $N_{p}$ is the number of antenna ports. This precoding matrix maps data layers onto the number of antenna ports. After the multiplication with the matrix $W_{\mathrm{BB}}$, the resulting data symbols are transposed. Then, the precoded and transposed symbols are passed through a subcarrier mapping matrix $T\in C^{N\times N_{d}}$, being *N* the number of subcarriers per OFDM symbol. Finally, the output of the matrix T is converted to the time domain via $F^{H}$ where $F^{H}\in C^{N\times N}$ is an inverse discrete Fourier transform (IDFT). The final signal $x\in C^{N\times N_{p}}$ in the time domain is generated as following: $x=F^{H}T{\left(\mathrm{Wd}\right)}^{T}$. Afterward, a CP is added to the resulting time signal before being fed into the non-linear PA.

Regarding the DFT-S-OFDM signal generation (Figure 1), data symbols are mapped onto a DFT matrix denoted by $D\in C^{M\times M}$ via a mapping matrix $M_{t}\in C^{M\times N_{d}}$, where *M* is the DFT size. Then, the output of the DFT is mapped onto a set of subcarriers in the frequency domain through another mapping matrix $M_{f}\in C^{N\times M}$. Finally, the output of the matrix $M_{f}$ is converted to time domain via $F^{H}$, where $F^{H}\in C^{N\times N}$ is the IDFT matrix and *N* is the number of subcarriers. The final signal $x\in C^{N\times 1}$ in the time domain is generated as follows: $x=F^{H}M_{f}DM_{t}d$. Then, a CP is added to the resulting time signal before being fed into the non-linear PA.

In both cases, output signals are amplified by a non-linear PA, which is assumed to be memory-less with amplitude-to-amplitude distortion only. In particular, a Rapp model is considered [26], whose amplitude-to-amplitude conversion function is given by the following:

(1) $$g\left(v,A\right)=vA{\left(1+\mathrm{abs}{\left(\frac{vA}{A_{\mathrm{sat}}}\right)}^{2p}\right)}^{\frac{-1}{2p}},$$

where *v* is the gain of the small signal, *A* is the amplitude of the input signal, $A_{\mathrm{sat}}$ is the limiting output amplitude, and *p* controls the smoothness of the transition from the linear region to the saturation regime. This model is widely used for OFDM/SC-FDMA systems to emulate the soft-limiting behavior of solid-state PAs. It captures AM/AM compression with a smooth transition to saturation. In our study, the Rapp model is applied to the UL transmitter chain of each UE to account for PA-induced distortion, which impacts the effective SNR observed at the receiver and, consequently, the performance of dynamic waveform switching.

A closed-loop power control mechanism is used in the UL [27], which considers that the BS decides a transmit power, $P_{\mathrm{tx}}$, to compensate for the path loss, PL. This transmit power can be expressed in decibels as $P_{\mathrm{tx}}=P_{0}+\alpha \mathrm{PL}$ dB, being $P_{0}$ a target received power at the BS, and α∈[0,1] the fractional compensation factor. An urban macro (UMa) model has been considered for computing the path loss component, PL.

Restrictions on the maximum transmit power supported by the UE and also out-of-band emissions, impose further limits on the transmitted power. The maximum power reduction (MPR) [1] specifies the decrease in the maximum power transmitted in order to enable the device to fulfill the requirements of the transmitter adjacent channel leakage ratio. This value imposes a maximum transmit power to guarantee that the out-of-band emission is below a given threshold. Since these out-of-band emissions depend on the waveform, modulation level and channel bandwidth, the possible MPR values also depend on such parameters. Finally, the final power transmitted by the UE, $P_{\mathrm{output}}$, can be defined as $P_{\mathrm{output}}=\min\left(P_{\mathrm{decided}},P_{\max}^{\prime }\right)$, where $P_{\max}^{\prime }=P_{\max}-\mathrm{MPR}$.

Table 1 summarizes the power reduction values as per the 5G specs. Notice that a higher maximum power can be used with DFT-S-OFDM, since its MPR is smaller than with CP-OFDM. This is an expected result as the former waveform is related to a smaller PAPR. DFT-S-OFDM has shown PAPR values between 7 and 8.5 dB while CP-OFDM produced values between 10 and 11 dB.

The SNR is computed as follows:

(2) $$\mathrm{SNR}=P_{\mathrm{output}}+G_{t}+G_{r}-\mathrm{PL}+10\;\log_{10}\left(\sum_{i=1}^{N_{\mathrm{rx}}}{\left|h_{i}\right|}^{2}\right)-N_{0}\;\left[\mathrm{dB}\right],$$

where $N_{\mathrm{rx}}$ is the number of receive antennas, and $h_{i}$ is the effective channel of the *i*-th receiver antenna after precoding (in case of CP-OFDM) and channel estimation. The thermal noise at the receiver at ambient temperature (i.e., 290*K*) is expressed as $N_{0}=-204+10\;\log_{10}\left(12\times \Delta f\times N_{\mathrm{RB}}\right)+N_{\mathrm{fig}}\;\;\left[\mathrm{dBW}\right]$, where Δf is the subcarrier spacing in Hz, $N_{\mathrm{RB}}$ is the number of PRBs assigned to the user and $N_{\mathrm{fig}}$ is the noise figure at the BS [28].

## 3. Dynamic Port and Waveform Switching

DPWS feature is targeted to switch between CP-OFDM and DFT-S-OFDM to enhance the UL coverage. It is designed to counteract the ping-pong effect that can occur when the signal rapidly switches back and forth, wasting network and device resources. To achieve that, the feature focuses on counting the so-called switching occasions before triggering the actual switching. The following parameters are involved:

- *Threshold* (ζ): it determines switching occasions from CP-OFDM to DFT-S-OFDM when the following inequality is fulfilled SNR<ζ.
- *Hysteresis* (ξ): it determines the switching occasions from DFT-S-OFDM to CP-OFDM as follows SNR>ζ+ξ.
- *Counter* (*C*): it counts for the number of switching occasions in order to trigger a waveform switching.
- *Timer* (*T*): it determines the time window to account for switching occasions. This time window is expressed as a number of SRS receptions.

The switching mechanism is influenced by two key factors: the estimated UL SNR (γ) per user, which is estimated periodically based on the SRS, and the current waveform, as follows. At a given time instant, if the transmission is being executed with CP-OFDM, then a switching occasion is counted if the SNR falls below ζ. However, if the transmission is being executed with DFT-S-OFDM, then a switching occasion is counted if the SNR rises above ζ+ξ. To trigger a waveform switch, the number of counted switching occasions must be equal to *C* within a time window that is smaller than *T*. The use of *C* and *T* on top of the commonly implemented hysteresis ξ offers a great stability against the ping-pong effect caused by small fluctuations in the signal. The switching mechanism is detailed in Algorithm 1, which is executed for each SRS reception.
**Algorithm 1** DPWS algorithm.**Input:** ζ, ξ, *C*, *T*, γ, CurrentWaveform**Initialize:** c=0, t=0  1: **if** (t<T) **then**  2:    **if** CurrentWaveform == CP-OFDM and γ<ζ **then**  3:        c=c+1
  4:    **else if** CurrentWaveform == DFT-S-OFDM and γ>ζ+ξ
  5:        c=c+1
  6:    **else**  7:        c=0,t=0
  8:    **end if**  9:    **if**
c≥C **then**
10:        Perform waveform and port switching11:    **end if**12:    t=t+1
13: **else**
14:    c=0,t=0
15: **end if**

Once the conditions of the algorithm have been met, the waveform switch is signaled to the UE via an RRC reconfiguration message, which changes the UL waveform from CP-OFDM to DFT-S-OFDM and vice-versa. Since the RRC reconfiguration message can change any parameter of the UL transmission, the 3GPP specifications reserve a guard time that allows the UE to prepare for transmission according to the new configuration. According to [27], this guard time depends on the numerology, μ, and on the UE category, but it ranges from 16.75 ms (μ=3, type 1 category) to 19 ms (μ=0, type 2 category). Therefore, each switch has a cost in performance since it involves an interruption in the UL transmission, which must be considered by the agent.

## 4. Proposed Deep Reinforcement Learning Dynamic Switching

Our proposed framework, AI-DWPS, is based on a DRL algorithm. In particular, a deep Q-learning (DQL) approach is used, where the q-table typically used in classical RL algorithms is substituted by a neural network called q-network. This q-network is implemented by a multi-layer perceptron (MLP) with a single hidden layer with $n_{\mathrm{hidden}}$ nodes and ReLU activation. The number of nodes in the input layer matches the cardinality of the state space, whereas the number of nodes in the output layer matches the cardinality of the action space. The output layer employs linear activation.

The proposed AI-DPWS framework optimizes the SNR threshold (ζ) and hysteresis (ξ) to maximize cell performance by means of a DRL-based mechanism. Its goal is to improve the throughput for cell-edge users, i.e., those with a lower CDF of the throughput, without reducing the overall average cell throughput. This dynamic adjustment is made automatically, which allows adapting the optimal thresholds to varying conditions of the environment. To this end, a reward function that encapsulates such a trade-off is devised. These parameters, SNR threshold (ζ) and hysteresis (ξ), are controlled by a DRL agent. The rest of the parameters are assumed to be fixed. The DPWS algorithm controls when and how the UL waveform and ports of the UE connected to the cell will switch.

The DPWS configuration of each cell can be changed based on the actions suggested by the DRL agent. The DRL is located in a non-real-time central network element (e.g., operations support systems (OSS) or an external server) and optimizes only the per-cell parameters (ζ,ξ). The gNB applies these parameters in its local DPWS loop; hence, the agent’s placement does not sit on the latency-critical path of per-UE waveform switching.

### 4.1. States, Actions, and Reward

#### 4.1.1. States

Given the nature of the problem, we have chosen to report statistics about two realistic uplink measurements available in actual networks: the SNR and the TA distributions. The throughput metric is defined as the rate of correctly decoded bits per second, whereas the TA indicates how distant the UE is from the serving BS. Both SNR and TA metrics are not reported on a per-connection (i.e., per-user) basis but as a histogram of all connections in a given time window. To ensure a seamless deployment in a real system, the bin´s limits have been inherited from the corresponding real-world KPIs in ERICSSON´s base stations. Each histogram is comprised of L=12 bins delimited to uniformly sample the typical range of operation of such KPIs. The SNR histogram has the following bins’ limits (in dB): [−∞,−5,−2,1,4,7,10,13,16,19,22,25,∞]; whereas, the bins’ limits for the TA histogram are (in %): [5,15,25,35,45,55,65,75,85,95,105,115,∞]. Note that the TA bins’ limits are expressed in terms of percentages, being 100% the TA associated with the propagation delay at the maximum range of the cell. This cell range is a network parameter representing the maximum distance (in meters) at which a given BS provides coverage. Due to the channel delay spread, the instantaneous measurement of the TA may provide a value above 100% for cell-edge users.

To compress the information given by these histograms we define the following descriptors for the TA and SNR metrics:

(3) $$R_{\ell ,\Psi }=\frac{\sum_{i=\ell }^{L}{\mathrm{BINS}}_{i,\Psi }}{\sum_{i=1}^{\ell -1}{\mathrm{BINS}}_{i,\Psi }},\;D_{\ell ,\Psi }=\frac{\sum_{i=\ell }^{L}{\mathrm{BINS}}_{i,\Psi }}{\sum_{i=1}^{L}{\mathrm{BINS}}_{i,\Psi }},$$

where Ψ stands for a given metric, i.e., either the SNR or TA, *L* is the number of bins, and ${\mathrm{BINS}}_{i,\Psi }$ stands for the number of UEs whose metric (TA or SNR) falls within the *i*-th bin interval, which is expressed as $\left[\psi_{i}^{-},\psi_{i}^{+}\right]$.

These metrics inform about the SNR and TA histograms while giving information about their shapes.

On the one hand, $R_{\ell ,\Psi }$ reflects the ratio of users above and below a certain threshold (bin edge), providing a sense of how many users are experiencing “good” vs. “bad” conditions. That is, $R_{\ell ,\mathrm{metric}}$ can be understood as the ratio between the number of occurrences of the random metric being above the smaller edge of the *ℓ*-th bin, $\psi_{\ell }^{-}$, and the number of occurrences below such edge of the *ℓ*-th bin. Therefore, it can be seen as an estimation of the following quotient of probabilities, $\hat{\mathrm{Pr}}\left(\Psi \geq \psi_{\ell }^{-}\right)/\hat{\mathrm{Pr}}\left(\Psi <\psi_{\ell }^{-}\right)$.

On the other hand, $D_{\ell ,\Psi }$ represents the complementary cumulative distribution function (CCDF) of the metric, estimating the proportion of users above a certain bin threshold. The number of bins and their limits determine the granularity of these metrics. For the SNR, the bins range from poor to excellent signal conditions (e.g., [−∞,−5,−2,1,…]), while for TA, bins correspond to proximity to the base station in terms of percentage of maximum cell range. High $D_{\ell ,\mathrm{TA}}$ values indicate many users near the cell edge, where DFT-S-OFDM might be preferred, whereas high $D_{\ell ,\mathrm{SNR}}$ suggests overall strong signal conditions, where CP-OFDM could be advantageous.

These metrics play a key role in the DRL state representation. For example: $D_{9,\mathrm{TA}}$ indicates the proportion of users near the cell edge. TA and SNR are directly measurable in real-world networks, making the proposed system deployable without additional hardware or modifications. Furthermore, these metrics help evaluate how well the switching mechanism adapts to different network scenarios.

Let S be the state space, with s[n]∈S the instantaneous state at step *n*. The instantaneous state for the DLR algorithm is defined as

(4) $$s[n]=\{\zeta \left[n\right],\xi \left[n\right],\bar{\gamma }\left[n\right],R_{6,\mathrm{SNR}},R_{5,\mathrm{SNR}},R_{6,\mathrm{TA}},R_{3,\mathrm{TA}},D_{5,\mathrm{SNR}}\},$$

where $\bar{\gamma }$ is the average SNR across the cell’s UEs and slots related to a given step, ζ[n] is the SNR threshold at step *n*, and ξ is the SNR hysteresis at step *n*.

#### 4.1.2. Actions

Let A be the action space, with a[n]∈A being the action chosen by the agent at step *n*. The action defines the decision made for each optimizable parameter: SNR threshold (ζ) and SNR hysteresis (ξ). Both parameters are subjected to three possible options: (i) decrease the value of the parameter by a certain step, (ii) keep the existing value, or (iii) increase value by the same step. The decrease/increase step is fixed to Δζ=1 dB and Δξ=0.5 dB. A is therefore comprised of nine possible actions. Note that the use of small changes of ζ and ξ reduces the impact of wrong decisions made by the agent and allows the smooth acquisition of optimal values through several iterations. During training, the action selection follows a decaying ϵ-greedy policy. The DQN agent selects selects a random action with probability ϵ and the action associated with the highest value outputted by the Q-Network with probability 1−ϵ. As training progresses, the value of ϵ is gradually reduced from $\epsilon_{0}$ towards $\epsilon_{\min}$ allowing the agent to exploit the knowledge it has acquired. During evaluation, ϵ is set to 0.

#### 4.1.3. Reward

To accurately capture the performance of the users in the cell at time step *n*, the reward function is composed of a set of *K* reward factors, $G_{k}\left[n\right],k\in \left[1,K\right]\subset N$, each one corresponding to a certain percentile, $p_{k}$, of the throughput of the cell.

The reward function at step *n* is given by

(5) $$\begin{array}{cc} r_{\mathrm{ini}}\left[n\right] & =\theta \cdot B\cdot {\left(\Delta G_{1}\left[n\right],\Delta G_{2}\left[n\right],...,\Delta G_{k}\left[n\right]\right)}^{t}, \end{array}$$

(6) $$\begin{array}{cc} \Delta G_{k}\left[n\right] & =\frac{G_{k}\left[n\right]-G_{k}\left[n-1\right]}{G_{k}\left[n-1\right]}, \end{array}$$

where the superscript *t* converts the vector formed by the relative gains $\Delta G_{k}$[n] of the reward factors *k* into a column, $B=\left(B_{1},..,B_{K}\right)$ is the vector of weights with length *K* and θ is a scale factor meant to expand the value range of the reward to help with training. As it can be observed, this reward function focus on throughput because it is an end-to-end metric that determines the quality of service of a given connection. In addition, since the aim is to improve the performance of cell-edge users, lower percentiles of the throughput are included in this reward function. Nevertheless, as explained further in Section 5, the switching mechanism greatly increases the gain of the lower percentiles compared to the average throughput gain. Therefore, a weighing vector $B=\left(B_{1},..,B_{K}\right)$ is needed to prevent that the agent takes actions that worsen the average throughput. Additionally, to help stabilize the training, the reward peak values are limited by the factor $r_{\mathrm{clip}}$ as follows:

(7) $$r\left[n\right]=\max(\min(-|r_{\mathrm{clip}}|,r_{\mathrm{ini}}\left[n\right]),\left|r_{\mathrm{clip}}\right|).$$

### 4.2. Deployment Considerations and Latency Budget

We distinguish a slow loop (policy update) and a fast loop (per-UE switching). The slow loop collects aggregated SNR/TA histograms over a measurement window and updates (ζ,ξ) per cell; it tolerates transport/processing latencies typical of OSS/edge deployments. The fast loop runs at the gNB per SRS occasion (2 ms in our setup) and, upon meeting the counter condition, commands an RRC reconfiguration that incurs the standardized guard time (from 16.75 ms to 19 ms).

Centralizing inference reduces per-site computation requirements and eases system-wide coordination. The signaling overhead is limited to (i) periodic upload of KPI histograms and (ii) infrequent parameter updates consisting of (ζ,ξ) pairs.

Placing the agent at the gNB removes the slow-loop transport latency but increases processing load at the gNB. It does not change the RRC guard time per switch. Since (ζ,ξ) evolve more slowly than the SRS-driven dynamics exploited by DPWS, we adopt centralized inference by default and maintain the latency-sensitive switching logic at the gNB.

### 4.3. Adaptation to Non-Stationary Environments and Mobility

The architectural separation between the fast and slow loops is intrinsic to AI-DPWS. The latency-critical fast loop, executed locally at the gNB on each SRS occasion, bases its decisions on instantaneous SNR and triggers the corresponding RRC reconfiguration. The slow loop, in contrast, updates only the cell-level parameters (ζ,ξ) from aggregated SNR/TA histograms and therefore operates on a non-real-time horizon. While the evaluation in Section 5 focuses on stationary users, this separation remains valid when mobility induces rapid changes, since the fast loop continues to handle per-UE dynamics while the slow loop tracks long-term trends.

The DRL state is constructed from histograms of SNR and TA, as defined in Section 4.1, which are periodically updated at each aggregation window. As such, mobility-induced shifts in the distributions are naturally reflected in the agent’s input state. In stationary scenarios, consecutive histograms remain similar, whereas in mobile scenarios they evolve more rapidly, which the agent can exploit when adjusting (ζ,ξ). As a possible extension, temporal indicators (e.g., deltas or divergences between consecutive histograms) or adaptive window lengths could be introduced to accelerate adaptation, but the baseline design already provides mobility awareness through its histogram-based representation.

The DPWS mechanism at the gNB incorporates counters and timers that bound the effective switching rate and prevent ping-pong. These safeguards ensure that the per-UE switching cost remains dominated by the standardized RRC guard time, regardless of how often the slow loop may update (ζ,ξ). Thus, even if parameter updates become more frequent under high mobility, the switching behavior remains controlled.

In the current implementation, the DRL agent is assumed to reside in a central entity (e.g., OSS). As a deployment option for highly mobile environments, the agent could alternatively be placed closer to the RAN (e.g., at the edge) to increase the frequency of (ζ,ξ) updates. The per-UE fast loop and its latency budget remain unchanged. This flexibility is not part of the present evaluation but represents a practical extension of the framework.

## 5. Simulation Results

The environment has been simulated using the 5G toolbox of MATLAB R2024b, whereas the DRL agent has been implemented in Keras and Tensorflow. The detailed parameters of the DRL training are summarized in Table 2. During the length of the transmission, the UEs are bounded to the DPWS following the procedure described in Algorithm 1 according to the values of ζ and ξ of the step. UEs are also subjected to adaptive modulation and coding (AMC); therefore, the employed MCS is susceptible to change as the SNR evolves. At any given slot, UEs reporting SNR values below the threshold of the lowest MCS will fall into outage. During CP-OFDM transmissions, the precoding matrix W is selected by the BS from the corresponding codebook according to the SRS signal received in order to maximize the received SNR. RL-related parameters can be found in Table 2. All UEs share the same configuration parameters, which are summarized in Table 3. As defined in that table, SRS periodicity is set to 2 ms by default. DRL steps correspond to consecutive windows over which histograms are computed and (ζ,ξ) may be updated; they are not per-SRS decisions. Per-UE switching continues to be decided at the gNB at SRS cadence, and any actual waveform change includes the RRC guard time described in Section 3.

UEs resulting in outage for the complete duration of the transmission were not scheduled thus not considered in the derived metrics. Within a given episode, the UEs do not change across steps and their channel realizations are repeated. Said condition implies that observed changes in the throughput values can then only be induced by tweaking the parameters ζ and ξ via the RL agent.

The baseline simulation setup fixes the user speed to 0.4 km/h (stationary), as listed in Table 3, in order to isolate the effect of learning (ζ,ξ) without confounding mobility factors. Nevertheless, as discussed in Section 4.2, the fast loop at the gNB executes at every SRS occasion and remains latency-bounded by the standardized RRC guard time, independently of where the DRL agent resides. Furthermore, as detailed in Section 4.3, the use of periodically updated SNR/TA histograms already provides a degree of mobility awareness in the DRL state, since distribution shifts are naturally reflected across consecutive windows. In scenarios with higher mobility, the same architectural separation, stability safeguards, and possible extensions (e.g., temporal indicators or adaptive windowing) enable the framework to adapt to non-stationary conditions.

To accurately capture the performance in the edge cell, the reward includes *K* reward factors, $G_{k}$, associated with low throughput percentiles and the average throughput. Table 4 summarizes all selected reward factors $G_{k}$ and their associated weights $B_{k}$. It was observed during training that relative gains in the lower percentiles were disproportionately bigger compared to those closer to the median. In accordance with Equation (6), small values of $G_{k}\left[n-1\right]$ can result in $\Delta G_{k}\left[n\right]$ becoming large, even if the particular reward factor *K* changes slightly. This is especially true for the lowest percentiles. Such disparity between different values of $\Delta G_{k}\left[n\right]$ resulted in some reward factors dominating the reward while others became completely obscured, effectively producing no meaningful variations in the overall reward function r[n]. This over-representation of lower percentiles in the reward function caused the agent to bias its behavior towards excessively enhancing the edge users while seriously damaging mid-tier and average users´ performance. This bias is mitigated with a vector **B** that amplifies the $\Delta G_{k}\left[n\right]$ of higher percentiles. The final configuration of linearly increasing weights in Table 4 was selected empirically. Other configurations of reward factors and weights were also tested with under-performing results.

After the training stage was completed, the agent was evaluated on 15 different episodes. Given the incremental nature of the action space, the agent was given 15 steps to reach the solution for each episode.

Table 5 summarizes the achieved average throughput gains of the AI-DPWS in contrast to fixed waveform schemes. Throughput gains are expressed in relative terms for all reward factors $G_{k}$. These results are drawn from the cumulative metrics of all UEs in the 15 evaluation episodes. The proposed scheme consistently outperforms the case of using any of both waveform schemes. This improvement is remarkable at lower percentiles, which is related to cell-edge users, achieving gains higher than 30%. Importantly, these gains at lower percentiles do not detrimentally impact the cell’s average throughput. Therefore, the fairness in the system is also improved since cell-edge users improve their performance without any penalty in average throughput.

At the same time, the relative gains in the p25–p45 range are smaller and in some cases slightly negative compared to CP-OFDM. This behavior is consistent with the design of the reward function (Equations (5) and (6)), which is formulated to produce larger relative gains in lower percentiles to explicitly bias the agent towards improving coverage-limited users. These mid-range trade-offs are bounded, as the inclusion of higher percentiles and the average throughput in the reward function acts as a safeguard. Median and aggregate performance values, therefore, remain close to the best fixed waveform scheme, while fairness is substantially improved by lifting the weakest users.

**Figure 2:**
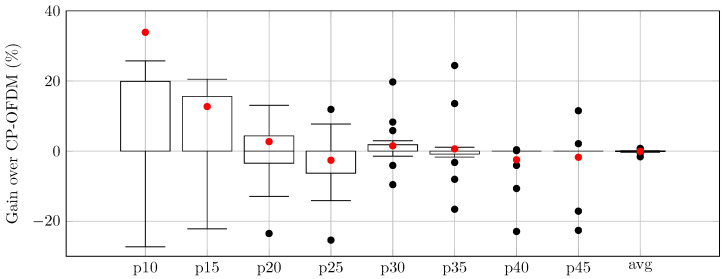
Relative gains of AI-DPWS over CP-OFDM in percentage.

**Figure 3:**
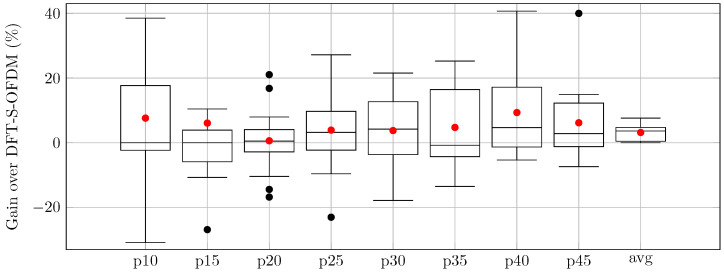
Relative gains of AI-DPWS over DFT-s-OFDM in percentage.

The relative throughput gains drawn as boxplots for the $G_{k}$ reward factors of the proposed AI-DPWS framework over CP-OFDM and DFT-s-OFDM are presented in Figure 2 and Figure 3, respectively. The red dots in each boxplot correspond to the average value, matching the values from Table 5.

**Figure 4:**
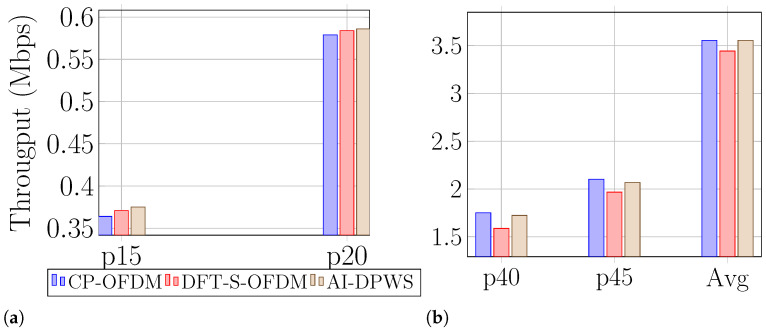
Achieved throughput with both fixed waveforms and with AI-assisted switching averaged over all episodes in evaluation for: (**a**) low percentiles and (**b**) high percentiles.

Finally, to better visualize the impact of AI-DPWS across all UEs in the evaluation, Figure 4a,b illustrate the achieved throughput of both fixed waveforms and AI-DPWS for different percentiles. As expected, for the lower percentiles, i.e., the cell-edge UEs, DFT-S-OFDM outperforms CP-OFDM. However, AI-DPWS outperforms both waveforms by taking into account the conditions of the channel and switching the waveforms accordingly. For percentiles closer to the median, CP-OFDM outperforms DFT-S-OFDM and in this case, AI-DPWS can only match the performance of the best fixed waveform scheme. It is worth noting that the silence periods caused by waveform switchings resulted in an average loss of 4.19% throughput across the evaluation episodes. This corresponds with a worst-case of 19 ms reconfiguration delay for μ=0 and type 2 UE category.

## 6. Conclusions

In this paper, a DRL-based framework is proposed to dynamically select the optimal threshold and hysteresis for UL waveform selection based on the DPWS scheme. By using a realistic 5G simulator and realistic measurements available in current networks, it is shown that the proposed scheme outperforms fixed schemes where the waveforms are selected in a static manner. Such performance improvements are exhibited across a wide range of throughput percentiles, showing bigger gains in the lower percentiles, which accounts for the cell-edge UEs. These improvements come without any harm to the average UEs in the cell.

Beyond this work, several future directions can be envisioned. First, the AI-DPWS framework could be extended to support reduced-capability (RedCap) devices and massive machine-type communication (mMTC) user types, which are becoming increasingly relevant in industrial and IoT applications. These categories introduce distinctive traffic patterns and constraints, such as sporadic transmissions, energy efficiency requirements, and limited processing power, which would test the flexibility of the framework in optimizing (ζ,ξ) under diverse conditions. Second, investigating the scalability of the method in multi-cell deployments is an important step to assess coordination overheads and system-wide efficiency. In such scenarios, the interaction between neighboring cells may require new strategies for distributed learning or parameter sharing to prevent instability while leveraging spatial diversity. Finally, further exploration of adaptation under mobility and sudden traffic variations would enhance the robustness of the framework for highly dynamic environments. While the current design already benefits from the separation of fast and slow loops and the use of histogram-based states, future work could evaluate adaptive windowing or temporal-difference features to improve responsiveness against abrupt changes in network load or user movement.

## Figures and Tables

**Table 1:** MPR values [1].

		MPR (dB)	
**Waveform**	**Modulation**	**50/100/200 MHz** **Channel BW**	**400 MHz** **Channel BW**
DFT-S-OFDM	Pi/2BPSK	1.5	3.0
	QPSK	1.5	3.0
	16QAM	3.0	4.5
	64QAM	5.0	6.5
CP-OFDM	QPSK	3.5	5.0
	16QAM	5.0	6.5
	64QAM	7.5	9.0

**Table 2:** RL parameter settings.

Parameter	Value
$\left[\epsilon_{0},\epsilon_{\min}\right]$	[1, 0.01]
Learning rate	0.05
Training steps per episode	75
# of training episodes	43
Evaluation steps per episode	20
# of evaluation episodes	16
UEs per episode	50
$n_{\mathrm{hidden}}$	60
Optimizer	Adam
Discount factor	0.01
Batch size	350
Experience buffer size	750
Default threshold, ζ (dB)	0
Default hysteresis, ξ (dB)	5
$\left(\theta ,r_{\mathrm{clip}}\right)$	(50,2)

**Table 3:** Network parameter settings.

Parameter	Value
$N_{\mathrm{RB}}$ (RBs)	20
Transmission length (slots)	1000
Carrier frequency (GHz)	28
Subcarrier Spacing (kHz)	15
User speed (km/h)	0.4 (stationary)
Delay Spread (ns)	30
Channel Delay Profile	CDL-A
Pathloss model	Urban Macro
$G_{t}$ (dB)	13.7
$G_{r}$ (dB)	20.6
SRS Periodicity (ms)	2
Min/max UE to BS distance (m)	25/300
$\left(A_{\mathrm{sat}},p\right)$	(24 dBm, 2)
$P_{\max}$ (dBW)	−7
RRC3econfiguration delay	19 ms

**Table 4:** Reward factors and their corresponding weights.

$G_{k}$	p10	p15	p20	p25	p30	p35	p40	p45	Avg
** $B_{k}$ **	0.02	0.04	0.06	0.08	0.10	0.12	0.14	0.16	0.18

**Table 5:** Evaluation results.

	Gains Over: (%)	
$G_{k}$	**CP-OFDM**	**DFT-S-OFDM**
p10	33.647	7.407
p15	11.023	5.576
p20	3.220	0.675
p25	−2.602	4.220
p30	1.747	4.046
p35	−0.142	4.123
p40	−2.475	9.325
p45	−2.316	5.549
Avg	−0.049	3.014

## Data Availability

The data presented in this study are available on request from the corresponding author.
